# Assessing forces during spinal manipulation and mobilization: factors influencing the difference between forces at the patient-table and clinician-patient interfaces

**DOI:** 10.1186/s12998-020-00346-1

**Published:** 2020-11-10

**Authors:** Jérémie Mikhail, Martha Funabashi, Martin Descarreaux, Isabelle Pagé

**Affiliations:** 1 Department of Chiropractic, Université du Québec à Trois-Rivières, 3351 boul. Des Forges, Trois-Rivières, G8Z 4M3 Québec Canada; 2 Division of Research and Innovation, Canadian Memorial Chiropractic College, 6100 Leslie St, Toronto, Ontario M2H 3J1 Canada; 3 Department of Human Kinetics, Université du Québec à Trois-Rivières, 3351 boul. Des Forges, Trois-Rivières, G8Z 4M3 Québec Canada; 4 Center for Interdisciplinary Research in Rehabilitation and Social Integration (CIRRIS), 25 Wilfrid-Hamel Blvd., Québec, G1M 2S8 Québec Canada

**Keywords:** Force, Kinetics, Spinal manipulative therapy, Thoracic spine

## Abstract

**Background:**

Spinal manipulative therapy (SMT) and mobilization (MOB) effects are believed to be related to their force characteristics. Most previous studies have either measured the force at the patient-table interface or at the clinician-patient interface. The objectives of this study were to determine 1) the difference between the force measured at the patient-table interface and the force applied at the clinician-patient interface during thoracic SMT and MOB, and 2) the influence of the SMT/MOB characteristics, participants’ anthropometry and muscle activity (sEMG) on this difference.

**Methods:**

An apparatus using a servo-linear motor executed 8 SMT/MOB at the T7 vertebrae in 34 healthy adults between May and June 2019. SMT and MOB were characterized by a 20 N preload, total peak forces of 100 N or 200 N, and thrust durations of 100 ms, 250 ms, 1 s or 2 s. During each trial, thoracic sEMG, apparatus displacement as well as forces at the patient-table interface and the clinician-patient interface were recorded. The difference between the force at both interfaces was calculated. The effect of SMT/MOB characteristics on the difference between forces at both interfaces and correlations between this difference and potential influencing factors were evaluated.

**Results:**

Force magnitudes at the patient-table interface were, in most trials, greater than the force at the clinician-patient interface (up to 135 N). SMT/MOB characteristics (total peak force, thrust duration and rate of force application) affected the difference between forces at both interfaces (all *p*-values< 0.05). No factor showed significant correlations with the difference between forces at both interfaces for the 8 SMT/MOB.

**Conclusions:**

The results revealed that the force measured at the patient-table interface is greater than the applied force at the clinician-patient interface during thoracic SMT and MOB. By which mechanism the force is amplified is not yet fully understood.

## Introduction

Spinal manipulative therapy (SMT) and spinal mobilization (MOB) are commonly used by several health care professionals in the management of musculoskeletal conditions [1]. SMT is characterized by the application of a dynamic force using a high-velocity and low-amplitude thrust, whereas MOB is defined by the application of a cyclic and rhythmic low-velocity force to the intervertebral joint. Forces, from both interventions, cause a mechanical deformation of the spinal region and surrounding tissues and are believed to trigger neuromechanical responses that potentially contribute to their respective therapeutic effects [2–4].

The quantification of SMT and MOB forces has been the focus of several studies and is fundamental to better understanding the underlying biomechanical mechanism in which SMT and MOB act on the body. Although previous studies have investigated the forces applied during SMT and MOB, most studies have focused on the characteristics of the reaction forces (at the patient-table interface) or forces directly applied to the patient (at the clinician-patient interface) [5–7]. A systematic review by Downie et al. (2010) showed that forces measured at the patient-table interface reach on average 1044 N (±186 N) and that applied peak forces at clinician-patient interface during thoracic SMT vary between 238 N and 561 N [8]. For MOB, a systematic review by Snodgrass et al. (2006) showed that forces vary greatly as a function of the mobilization grade, with measured forces at the patient-table interface during thoracic MOB grade IV ranging from 232 N to 500 N [7].

Nevertheless, to elucidate SMT and MOB biomechanical underlying therapeutic mechanisms, it is fundamental to understand all forces acting on the body during the application of these interventions and how they interact with each other. Forces at both the patient-table interface and the clinician-patient interface have only been measured concomitantly by Kirstukas & Backman (1999) [5]. Based on mathematical models and given the deformable behaviour of the human body during dynamic loading application, forces at the patient-table interface were expected to be larger than the forces applied at the clinician-patient interface. Results from this study, however, showed that the peak forces at the patient-table interface were, on average, 16% lower than the peak forces at the clinician-table interface. Limitations related to the measurement instruments used in the study were outlined by the authors.

New investigative tools are now available, and limitations reported previously can now be addressed. Specifically, at the clinician-patient interface, a servo-controller linear actuator motor capable of applying SMT and MOB with repeatable and standardized forces was developed [9], significantly advancing the investigation of SMT and MOB applied forces (ex. Pagé et al. 2014;2018 [10, 11] and Nougarou et al. 2013;2014 [12, 13]). On the other hand, the Force Sensing Table Technology (FSTT®) accurately measures the forces at the patient-table interface [14]. By combining these two technologies, more accurate measurements and comparisons of forces at both interfaces during SMT and MOB are possible. Furthermore, other factors such as the technique parameters (total peak force magnitude and rate of force application) and the person receiving the SMT or MOB (age, percentage body fat, thorax thickness and muscle activation during technique application) can potentially influence the magnitude of the difference between forces at both interfaces during SMT and MOB. A better understanding of such interplay will further significant knowledge related to SMT and MOB biomechanics and potentially reveal important information related to underlying physiological mechanisms of manual therapy. Moreover, the results could guide the development of futures studies assessing SMT/MOB characteristics.

Therefore, this study aimed to 1) quantify the difference between the forces measured at the patient-table interface and the force applied at the clinician-patient interface during standardized thoracic SMT and MOB in asymptomatic adults and 2) to explore the factors related to SMT/MOB force characteristics and participant characteristics that potentially influence the magnitude of this difference. Based on the deformable behaviour of the human body and the viscoelastic properties of biological tissues, it was hypothesized that the forces measured at the patient-table interface would be greater than the force applied at the clinician-patient interface. It was also hypothesized that the difference in force between both interfaces would vary as a function of the SMT/MOB characteristics, muscle activation, and participant’s body composition.

## Methods

### Participants

Adults without thoracic pain and aged between 18 and 50 years old were recruited through advertisement on social media and word of mouth between May and June 2019. Participants were excluded if they presented any contraindication to SMT and MOB [15], had a history of spine surgery, vertebral fracture or were diagnosed with a spine infection, osteopenia or thoracic scoliosis. The study was approved by the Université du Québec à Trois-Rivières human research ethics committee (CER-19-257-07.19) and all participants provided their written informed consent prior to participating in the study.

### Protocol summary

Participants were invited to take part in two 60-min experimental sessions conducted 2 to 4 days apart. During the first sessions, participants’ age, sex and anthropometry (height, weight, thorax thickness and percentage of body and trunk fat) were obtained.

Participants were asked to lie prone on a force-sensing table technology (FSTT®, detailed below) and T5, T6 and T7 spinous processes were identified by palpation. T7 transverse processes were also identified using the landmarks suggested by Cooperstein et al. (2009) [16] and Pagé et al. (2017) [17]. Surface electromyography (EMG) electrodes were then placed on the skin overlying the thoracic erector spinae (TES) muscles, and a normalization trial was performed. While the participant was lying in prone position, two 1.14 kg weights were placed on a wood stool on each side of the participant’s head. Participants were then instructed to take a weight in each hand and lift them just over the support and hold for 5 s while EMG signals were recorded.

The apparatus used to deliver SMT and MOB (detailed below) was then positioned on the participant’s back with its rod tip aligned with T7 transverse processes. T7 was chosen for all participants to limit the impact of the spine curvature as it has been described to be commonly the apical vertebrae of the thoracic kyphosis [18]. A total of four SMT and four MOB with different force-time characteristics were delivered over the two sessions using a randomized order (four per session). During each technique application, muscle activity, rod displacement, forces at the patient-table interfaces and the apparatus-patient interface (further referred as the clinician-patient interface to ease comparison with previous literature) were respectively recorded by the surface EMG electrodes, the FSTT® and the apparatus.

### Instrumentation

#### Anthropometric measurements

Height was measured using a tape mounted on the wall while weight and percentage of body and trunk fat was assessed using a bioelectrical impedance scale (Segmental Body Composition Analyzer, Tanita BC-418). A caliper (S&S X-Ray Products Inc. Brooklyn, NY, error ± 0.5 cm) was used to measure the thorax thickness, defined as the distance between the skin at the level of T7 spinous process and the surface of the FSTT® when the participant was lying in prone.

#### Force-sensing table

A force-sensing treatment table was used to measure the forces at the anterior aspect of the thorax (patient-table interface) during each SMT and MOB. The Force Sensing Table Technology (FSTT®, Toronto, Ontario, Canada) is composed of a treatment table and an integrated AMTI force plate (Advanced Mechanical Technology Inc., Watertown, Massachusetts, USA). The FSTT® has been shown to be reliable in the measurement of SMT force-time characteristics [14]. The 3-dimensional force plate voltages in Fx, Fy and Fz were recorded at 1 kHz with a 12-bit A/D converter.

#### Surface electromyography (EMG)

Four bipolar surface EMG electrodes (10 mm interelectrode distance, Delsys, Inc., Boston, MA) were used to record TES muscle activity during each SMT and MOB application. Electrodes were applied bilaterally at approximately 2 cm laterally of the T5 and T7 spinous processes in line with TES muscle fibers. A reference electrode was applied on the left lateral malleolus. Prior to electrode placement, the skin was shaved, slightly abraded and cleaned with alcohol swabs to reduce impedance. Data were recorded at 1 kHz with a 12-bit A/D converter. EMG and forces (at the clinician-patient and patient-table interfaces) data acquisitions were synchronized.

#### Manual technique application

An apparatus using a servo-controlled linear actuator motor (Linear Motor Series P01–48 × 360, LinMot Inc., Zurich, Switzerland) was used to deliver standardized SMT and MOB. This apparatus has both high repeatability and precision in delivering standardized SMT and MOB forces [9]. The apparatus indenter consisted of a twin-tip padded rod (θ tip =10 mm; distance between the center of the tips =56 mm) positioned on the skin overlying the T7 transverse processes. Accurate re-positioning of the device was ensured by marking the location of the twin-tip with an ink pen at the end of the first session.

**Fig. 1:**
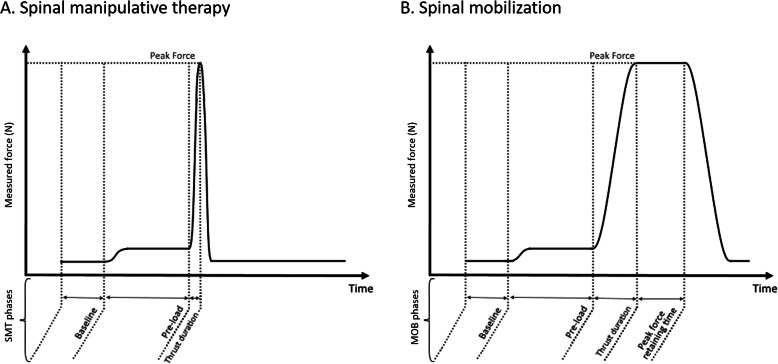
Force-time graphs of a typical (**a**) spinal manipulative therapy and (**b**) spinal mobilization

Figure 1 shows examples of typical SMT and MOB force-time graphs, while Table 1 presents the force-time characteristics of the four SMT and four MOB applied in the current study. The force-time characteristics used in this study were based on data reported in the literature [7, 8]. Specifically, SMT and MOB were characterized by a 20 N preload force maintained during 1 s, followed by a total peak force of 100 or 200 N. For SMT, thrust durations were of 100 or 250 ms and the total peak force was immediately removed once reached. For MOB, thrust durations were of 1 or 2 s and the total peak force was maintained for the same duration as the thrust duration before being removed. The displacement of the twin-tip indenter (mm) and the force (N) generated by the apparatus during the SMT and MOB were recorded using LinMot-Talk® software (version 5.1, LinMot Inc., Zurich, Switzerland) at a frequency of 256 Hz.

**Table 1:** Biomechanical characteristics of the four SMT and four MOB

Force-time profiles	Preload force (N)	Total peak force (N)	Thrust duration (ms)	Total peak force retaining time (ms)	Rate of force application (N/s)
SMT1	20	100	100	0	800
SMT2	20	100	250	0	320
SMT3	20	200	100	0	1800
SMT4	20	200	250	0	720
MOB1	20	100	1000	1000	80
MOB2	20	100	2000	2000	40
MOB3	20	200	1000	1000	180
MOB4	20	200	2000	2000	90

Both SMT and MOB were performed 5 min apart during which participants rested quietly on the treatment table. To avoid soreness and tiredness, the eight applications were delivered over two sessions. The order of the force-time characteristics was randomly determined to minimize any sequential effect. At the start of the second session (2 to 4 days later), absence of pain in the thoracic region was confirmed by asking the participants to complete a 11-point visual analog scale for the assessment of pain [19]. If a score of 2 or greater was noted, the session was postponed for 24 h.

### Data processing and analysis

#### Calculation of the difference in force between interfaces

**Fig. 2:**
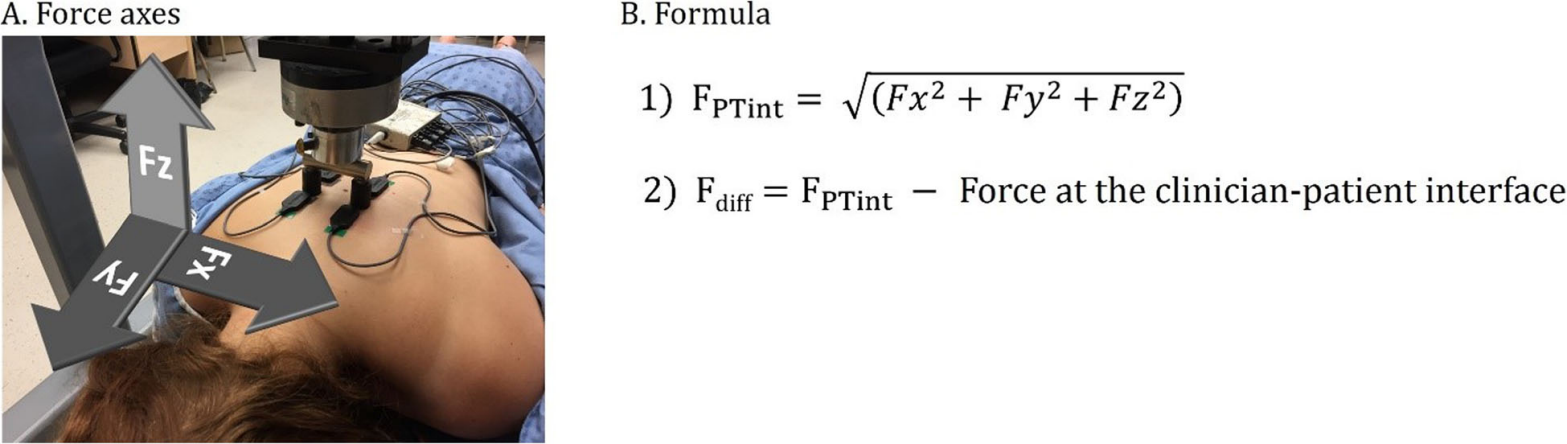
**a**. Apparatus used to deliver SMT and MOB, surface EMG electrodes recording muscle activity and schematization of the FSTT® coordinate system. **b**. Formula to calculate the difference in force between the patient-table and the clinician-patient interfaces (F_diff_)

For each trial, the difference of force between the preload and the total peak force was calculated for data measured at both the patient-table interface (FSTT® table data) and the clinician-patient interface (apparatus data). Since the FSTT® measures forces in the three axes of motion (Fx, Fy and Fz), the overall force at the patient-table interface (F_PTint_) was calculated. The difference in the force obtained at both interfaces (F_diff_) was then computed with a positive F_diff_ corresponding to a greater force at the patient-table interface than the applied force at the clinician-patient interface. The FSTT® coordinate system and F_diff_ formula are respectively presented in Fig. 2a and b.

#### EMG data processing

EMG data were first digitally band-pass filtered (20–450 Hz). For each SMT and MOB, the surface EMG signal was synchronized with the apparatus’ force data to determine the time-window from the start of the thrust to the total peak force. Muscle activity amplitude (root mean square, RMS) was then calculated for the four surface EMG electrodes and each electrode’s RMS value was further divided by its respective value obtained during the normalization trial. The left and right normalized RMS value (nRMS) of electrodes located at the same spinal level were then averaged (T5 nRMS and T7 nRMS) and used for subsequent analyses.

#### Statistical analysis

##### Main analysis

A descriptive analysis of the participants’ characteristics was computed. Mean (SD) values are reported for parametric data and median with inter-quartile range (IQR) for non-parametric data.

A mixed-model ANOVA was computed to assess the effects of the total peak forces (2 levels) and the thrust durations (4 levels) on F_diff_. When indicated, the Tukey post-hoc test was computed to depict the significant differences. A repeated measures ANOVA with planned comparisons for linear trend was also conducted to assess the effect of the rate of force application (8 levels) on F_diff_.

##### Exploratory analysis

For each SMT and MOB force-time profile (Table 1), exploratory correlations between F_diff_ and potential factors influencing this difference were computed. Factors included the apparatus rod displacement, participants’ anthropometry (weight, height, body mass index [BMI], percentage of body and trunk fat, and thorax thickness) and muscle activity amplitude (T5 nRMS and T7 nRMS). Pearson’s correlation coefficient was computed when the factor presented a parametric distribution, while Pearson’s estimated value obtained from Kendall Tau B correlation coefficients was computed for nonparametric data distribution. The strength of the correlations was evaluated as “strong” (r ≥ 0.70), “good” (0.50 ≤ r ≤ 0.70), “moderate” (0.30 ≤ r ≤ 0.50), or “poor” (r ≤ 0.30) [20]. Finally, t-tests for independent samples were used to assess whether F_diff_ was significantly different between males and females.

Statistical analysis was conducted using Statistica™ (Version 13.3, TIBCO software Inc., USA) with a statistical significance set at *p <* 0.05.

## Results

### Participants

Of the 35 participants recruited, one was excluded following the first SMT trial due to pain during the procedure. Data from one additional participant were excluded as it was incomplete. The data of the remaining 33 participants were used for the analyses (Table 2).

**Table 2:** Participants characteristics

Characteristics	Value (***n*** = 33)
Females: Males	18: 15
Age (years; mean ± SD)	24.15 ± 2.70
Weight (kg; median ± IQR)	70.00 ± 20.70
Height (m; median ± IQR)	1.69 ± 0.11
BMI (kg/m^2^; median ± IQR)	24.00 ± 4.00
Percentage body fat (%; median ± IQR)	20.00 ± 15.30
Percentage trunk fat (%; median ± IQR)	19.10 ± 11.15
Thorax thickness (cm; median ± IQR)	19.00 ± 2.00

### Difference in force between both interfaces

The mean (SD) values of the F_diff_ are reported in Table 3 for each force-time profile. For most trials, greater forces at the patient-table interface than at the clinician-patient interface were measured.

**Table 3:** Difference between forces at the patient-table interface and the clinician-patient interface (F_diff_) for each SMT force-time profile

Force-time profiles^a^	F_**diff**_			Participants showing greater force at the patient-table interface (n)	Participants showing greater force at the clinician-patient interface (n)
	Mean^b^	SD	Range		
SMT1	15.56 N	4.86 N	5.35 N to 26.09 N	33	0
SMT2	4.74 N	6.72 N	−7.35 N to 29.06 N	26	7
SMT3	36.70 N	10.02 N	10.45 N to 56.82 N	33	0
SMT4	16.74 N	14.13 N	−8.41 N to 68.04 N	31	2
MOB1	8.77 N	12.34 N	−11.22 N to 57.82 N	26	7
MOB2	7.12 N	10.74 N	−8.58 N to 47.10 N	26	7
MOB3	29.23 N	28.55 N	−4.28 N to 135.08 N	29	4
MOB4	21.40 N	25.24 N	−11.39 N to 130.05 N	30	3

^a^ The list refers to the force-time profiles presented in Table 1

^b^ A positive F_diff_ denotes that the force measured at the patient-table interface was greater than the force measured at the clinician-patient interface

### Effects of the SMT and MOB characteristics

Mixed-model ANOVAs showed that the thrust duration (F_3,96_ = 13.09, *p* < 0.001, ${\eta}_p^2$ =0.29) and the total peak force (F_1,32_ = 92.73, *p* < 0.001, ${\eta}_p^2$ =0.74) significantly affect F_diff_. The interaction effect between the thrust duration and the total peak force was also significant (F_3,96_ = 3.44, *p* = 0.02, ${\eta}_p^2$ =0.10). Results of the Tukey post-hoc tests are presented in Table 4.

**Table 4:** Significant differences in F_diff_ between the force-time profiles

Force-time profiles	SMT2 (mean = 4.74 N)	SMT3 (36.70 N)	SMT4 (16.74 N)	MOB1 (8.77 N)	MOB2 (7.12 N)	MOB3 (29.23 N)	MOB4 (21.40 N)
**SMT1**	*p <* 0.001*	*p <* 0.001*	*p* ≥ 0.05	*p* ≥ 0.05	*p* = 0.02*	*p <* 0.001*	*p* ≥ 0.05
**SMT2**		*p <* 0.001*	*p <* 0.001*	*p* ≥ 0.05	*p* ≥ 0.05	*p <* 0.001*	*p <* 0.001*
**SMT3**			*p <* 0.001*	*p <* 0.001*	*p <* 0.001*	*p* ≥ 0.05	*p <* 0.001*
**SMT4**				*p* = 0.03*	*p* = 0.004*	*p <* 0.001*	*p* ≥ 0.05
**MOB1**					*p* ≥ 0.05	*p <* 0.001*	*p <* 0.001*
**MOB2**						*p <* 0.001*	*p <* 0.001*
**MOB3**							*p* = 0.04*

* significant differences at *p* < 0.05

**Fig. 3:**
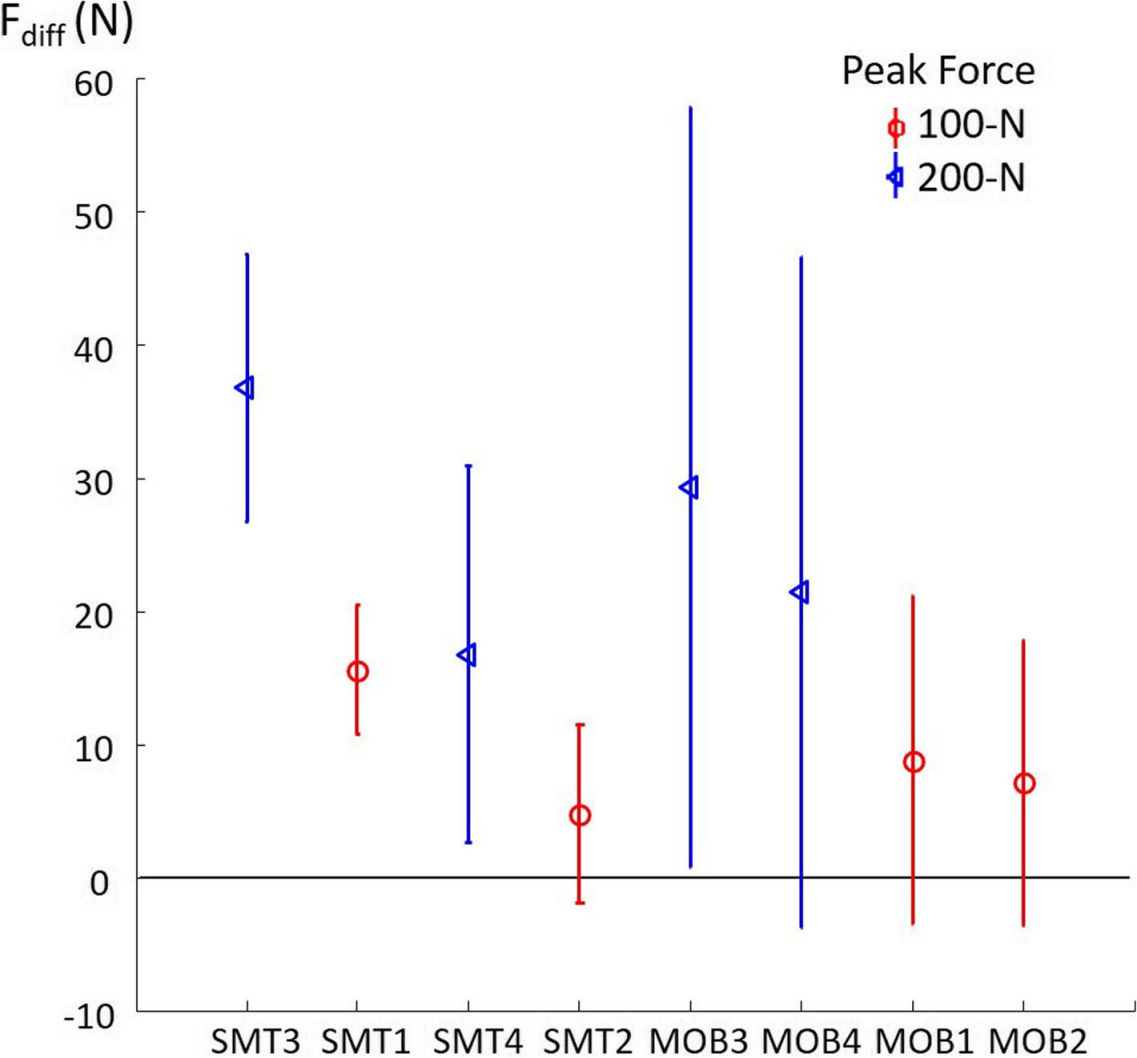
Mean (with SD) F_diff_ from the SMT with the higher rate of force application (SMT3) to the MOB with the lower rate of force application (MOB2). A significant linear trend was observed revealing a decrease in F_diff_ with the decrease in rate of force application (*p* < 0.0001)

Considering the significant interaction between the thrust duration and the total peak force on F_diff_, the rate of force application effect was analysed. The analysis revealed a significant rate of force application main effect (F_7,224_ = 24.61, *p* < 0.001, ${\eta}_p^2$ =0.43). Planned contrasts demonstrated a significant linear decrease in F_diff_ with the decrease in rate of force application (F_1,32_ = 28.65, *p* < 0.0001) (Fig. 3). Overall, the SMT with the higher rate of force application (SMT3: 1800 N/s) and the MOB with the lower rate of force application (MOB2: 40 N/s) resulted in a mean difference of 29.58 N (95% CI: 24.47–34.69 N) in F_diff_.

### Exploratory analysis

Table 5 presents the correlations between F_diff_ and potential influencing factors as well as the differences between males and females. A significantly greater F_diff_ was observed in females compared to males during SMT1 (T_32_ = 2.14, *p* = 0.04) with a mean difference of 3.35 N (95%CI = 0.16 to 6.54 N). Significant differences between females and males were not observed for the other force-time profiles (all *p* values > 0.05).

**Table 5:** Correlations^1^ between F_diff_ and potential influencing factors and differences between sex^2^

Factors	SMT1	SMT2	SMT3	SMT4	MOB1	MOB2	MOB3	MOB4
Sex	T_32_ *=* 2.14, *p =* 0.04*	T_32_ *=* 1.43, *p =* 0.16	T_32_ *=* 1.83, *p =* 0.08	T_32_ *=* 1.08, *p =* 0.29	T_32_ *=* 0.64, *p =* 0.53	T_32_ *=* 0.61, *p =* 0.55	T_32_ *=* 0.20, *p =* 0.84	T_32_ *=* 0.81, *p =* 0.42
Rod displacement	*r =* 0.67 *p =* 0.001*	r_e_ *=* 0.30 *p =* 0.09	*r =* 0.54 *p =* 0.001*	r_e_ *=* 0.05 *p =* 0.77	r_e_ *=* −0.12 *p =* 0.51	r_e_ *=* 0.11 *p =* 0.55	r_e_ *=* −0.17 *p =* 0.34	r_e_ *=* − 0.12 *p =* 0.49
Weight	r_e_ *=* −0.20 *p =* 0.26	r_e_ *=* − 0.27 *p =* 0.13	r_e_ *=* − 0.28 *p =* 0.11	r_e_ *=* − 0.32 *p =* 0.07	r_e_ *=* − 0.22 *p =* 0.21	r_e_ *=* − 0.24 *p =* 0.18	r_e_ *=* − 0.04 *p =* 0.82	r_e_ *=* − 0.08 *p =* 0.67
Height	r_e_ *=* − 0.31 *p =* 0.08	r_e_ *=* − 0.29 *p =* 0.10	r_e_ *=* − 0.34 *p =* 0.05	r_e_ *=* − 0.24 *p =* 0.19	r_e_ *=* − 0.12 *p =* 0.52	r_e_ *=* − 0.08 *p =* 0.67	r_e_ *=* 0.08 *p =* 0.66	r_e_ *=* 0.14 *p =* 0.43
Age	r_e_ *=* 0.18 *p =* 0.34	r_e_ *=* 0.16 *p =* 0.40	r_e_ *=* 0.05 *p =* 0.81	r_e_ *=* − 0.10 *p =* 0.58	r_e_ *=* − 0.29 *p =* 0.13	r_e_ *=* 0.17 *p =* 0.38	r_e_ *=* − 0.03 *p =* 0.89	r_e_ *=* 0.02 *p =* 0.91
BMI	r_e_ *=* − 0.05 *p =* 0.79	r_e_ *=* − 0.17 *p =* 0.33	r_e_ *=* − 0.14 *p =* 0.43	r_e_ *=* − 0.27 *p =* 0.13	r_e_ *=* − 0.23 *p =* 0.19	r_e_ *=* − 0.29 *p =* 0.10	r_e_ *=* − 0.12 *p =* 0.50	r_e_ *=* − 0.23 *p =* 0.21
Percentage of body fat	r_e_ *=* 0.33 *p =* 0.06	r_e_ *=* 0.23 *p =* 0.19	r_e_ *=* 0.27 *p =* 0.13	r_e_ *=* 0.21 *p =* 0.25	r_e_ *=* 0.09 *p =* 0.61	r_e_ *=* − 0.06 *p =* 0.75	r_e_ *=* − 0.03 *p =* 0.86	r_e_ *=* − 0.15 *p =* 0.39
Percentage of trunk fat	r_e_ *=* 0.34 *p =* 0.06	r_e_ *=* 0.19 *p =* 0.30	r_e_ *=* 0.25 *p =* 0.16	r_e_ *=* 0.16 *p =* 0.38	r_e_ *=* 0.09 *p =* 0.62	r_e_ *=* − 0.10 *p =* 0.57	r_e_ *=* − 0.05 *p =* 0.77	r_e_ *=* − 0.13 *p =* 0.46
Thickness	r_e_ *=* −0.01 *p =* 0.97	r_e_ *=* − 0.36 *p =* 0.049*	r_e_ *=* − 0.02 *p =* 0.90	r_e_ *=* − 0.21 *p =* 0.27	r_e_ *=* − 0.35 *p =* 0.06	r_e_ *=* − 0.53 *p =* 0.004*	r_e_ *=* − 0.19 *p =* 0.32	r_e_ *=* − 0.28 *p =* 0.13
T5 nRMS	r_e_ *=* − 0.03 *p =* 0.88	r_e_ *=* 0.07 *p =* 0.72	r_e_ *=* − 0.05 *p =* 0.80	r_e_ *=* 0.50 *p =* 0.01*	r_e_ *=* − 0.08 *p =* 0.70	r_e_ *=* 0.22 *p =* 0.26	r_e_ *=* 0.23 *p =* 0.22	r_e_ *=* 0.24 *p =* 0.20
T7 nRMS	r_e_ *=* 0.15 *p =* 0.44	r_e_ *=* 0.08 *p =* 0.67	*r =* −0.07 *p =* 0.71	r_e_ *=* 0.44 *p =* 0.02*	r_e_ *=* 0.07 *p =* 0.72	r_e_ *=* 0.15 *p =* 0.53	r_e_ *=* 0.42 *p =* 0.02*	r_e_ *=* 0.11 *p =* 0.56

* Significant correlation / difference at *p* < 0.05

^1^ Pearson correlation coefficient (r) or its estimated value from Kendall Tau rank coefficient (r_e_) are presented. Positive correlation value denotes an increase in F_diff_ with the increase in the factor value

^2^ Positive T-test value denotes greater F_diff_ in females

Only four factors showed significant correlations with F_diff_ and none presented consistent correlations among the eight force-time profiles. Apparatus’ displacement showed significant positive correlation with F_diff_ during the two fastest SMTs (SMT1, *r* = 0.67, *p* = 0.001; SMT3, r = 0.54, *p* = 0.001). Thoracic thickness was significantly correlated with F_diff_ during SMT2 (r_e_ = − 0.36, *p* = 0.049) and MOB2 (r_e_ = − 0.53, *p* = 0.004). Moreover, F_diff_ was significantly correlated with muscle activity during SMT4 (T5-nRMS, r_e_ = 0.50 *p* = 0.01; T7-nRMS, r_e_ = 0.44 *p* = 0.02) and MOB3 (T7-nRMS, r_e_ = 0.42, *p* = 0.02). No significant correlations between F_diff_ and the following factors were found for any of the force-time profiles: weight, height, age, BMI, percentage of body fat and percentage of trunk fat (all *p* values> 0.05).

## Discussion

In this study, the difference between the force measured at the patient-table interface and the force applied at the clinician-patient interface during thoracic SMT and MOB delivered was assessed in asymptomatic adults. The effect of different SMT and MOB force-time characteristics and potential influencing factors were also assessed. It was hypothesized that the forces measured at the patient-table interface would be greater than those measured at the clinician-patient interface and that SMT and MOB characteristics, muscle activity and anthropometric variables would influence the difference between forces at both interfaces.

In this study, the force applied at the clinician-patient interface was provided via a servo-controlled linear actuator apparatus, thus reducing variability previously observed by different hand configuration during force application [21, 22] and providing a systematic posterior to anterior force vector. Results showed that 93% of SMT and 84% of MOB yielded greater total peak forces at the patient-table interface. This was observed across the different SMT/MOB force-time profile. The average increase in force at the patient-table interface compared to the clinician-patient interface was 14.6%. There was also a significant effect of the SMT/MOB thrust duration and total peak force on the difference between forces at both interfaces. Consequently, the rate of force application also had a significant effect on the difference in forces indicating that lower rate of force application decreases the difference between the forces measured at the patient-table and clinician-patient interfaces.

Why do we measure increased forces at patient-table interface? In 1999, Kirstukas and Backman (1999) [5] were the first to record simultaneously the magnitude of the resultant force vector at the supporting patient-table interface and the perpendicular contact pressure distribution at the clinician-patient interface.. The authors hypothesized that due to the gravitational loads, clinician-applied loadings, and the accelerating patient’s mass, the patient-table interface force magnitude during SMT would be higher than the forces applied at the clinician-patient interface. However, their results showed the opposite as total peak forces at the clinician-patient interface exceeded total peak forces at the patient-table interface by 16%. The results of our study differ from those of Kirstukas and Backman (1999) [5], as forces recorded at the patient-table interface were generally higher than the forces applied at the clinician-patient interface by the apparatus. Not only do they differ, but they seem to support the hypothesis that near-static loading of the spine (MOB) yields different force patterns than higher rate of force application procedures (SMT). Indeed, differences between forces recorded at the patient-table interface and forces applied at the clinician-patient interface are much lower when lower rates of force application are used.

How do SMT/MOB force-time profile alter the difference between the force at the patient-table and the clinician-patient interfaces? Rate of force application and other SMT/MOB characteristics can modulate mechanical and neurophysiological responses generated during and following the technique application. For instance, the muscular response amplitude seems to increase with higher total peak force [12, 23, 24]. When thrust duration and total peak force were modulated while keeping a constant rate of force application, there was no difference observed in muscular response amplitude [25]. In animal studies, muscle spindle activity was amplified by either shorter thrust duration or higher total peak force during SMT [26–31]. Some animal studies even showed both, corresponding to a higher rate of force application [26–28]. Vertebral displacement also seems to be modulated by different SMT characteristics. Greater vertebral displacement is associated with increased total force applied at the clinician-patient interface [23, 25, 32]. Greater absolute vertebral displacement and lower relative displacement (i.e. displacement between two adjacent vertebrae) are also observed when MOB thrust durations are compared to SMT’s thrust durations [10]. In the present study, significant changes in the differences in forces at the patient-table and clinician-patient interfaces were observed when rate of force application was modulated. The observed changes support the hypothesis that higher rates of force application increase the difference between the forces at patient-table and clinician-patient interfaces.

None of the measured potential influencing factors was found to be associated with the difference between the forces at the clinician-patient and patient-table interfaces through all the eight SMT and MOB force-time profiles. Sex of the participant, apparatus displacement, thoracic thickness and muscle activity did show a significant correlation through only one or two set of SMT and/or MOB force-time profiles. These results did not confirm our initial hypothesis proposing that anthropometry would influence the difference between the forces at the patient-table and clinician-patient interfaces [33, 34].

### Strengths and limitations

The main strength of this study is the more precise measurement of the forces at both the patient-table and clinician-patient interfaces. Moreover, the use of an apparatus to deliver the SMT and MOB force-time profiles allowed the evaluation of the effect of the SMT and MOB characteristics on the difference between the forces at both interfaces. Limitations include the sample size limited to 33 participants as well as the used of a convenient sample of healthy young adults without spine related pain. Therefore, results might not apply to people with thoracic pain, pediatric patients or older adults. Considering the exploratory nature of the correlation analyses, corrections for multiple analyses were not computed. The observed significant correlations should therefore be interpreted with cautious. While a similar study with SMT and MOB being applied by a clinician has been conducted (manuscript under review), the extrapolation of this study’s results is currently limited. Indeed, manual therapy application configuration has been shown to influence the magnitude, location, and distribution of the pressure generated [35]. Consequently, the current study results might not be representative of manually applied SMT/MOB or of apparatus-based studies using other type of application.

### Clinical implications

The exploratory nature of this study limits the application of our results into clinical practice at this point. While results from this study suggest that anthropometric characteristics and muscle activation do not influence on the difference between the forces at the patient-table and clinician-patient interfaces, other potential influencing factors such as kyphosis degree and tissue stiffness should be investigated. Indeed, it has been described that structures with higher stiffness stress-shield adjacent tissues during movement [36] and future studies are planned to elucidate this. Most importantly, this study highlights the significance of the specific methods to measure the force-time characteristics during manual therapies as well as the limitations of comparing studies that use different methods of force measurement. Consequently, comparing manual therapy force-time characteristics measured at the patient-table interface with the ones measured at the clinician-patient interface is not recommended.

## Conclusion

This study revealed that, during thoracic manual therapy (SMT and MOB), forces measured at the patient-table interface are most often greater than forces applied at the clinician-patient interface. Moreover, manual therapy characteristics (total peak force, thrust duration and rate of force application) influence the difference of forces between the interfaces. Whether individual characteristics influence the transmission of force still needs to be further investigated. The results therefore suggest that manual therapy force-time profiles measured at the patient-table interface should not be compared to profiles measured at the clinician-patient interface.

## Data Availability

The datasets used and analysed during the current study are available from the corresponding author on reasonable request.

## Abbreviations

BMI: Body mass index
EMG: Electromyography
F_diff_: Difference between forces at the patient-table interface and at the clinician-patient interface
F_PTint_: Force at the patient-table interface
FSTT®: Force sensing table technology
IQR: Inter-quartile range
MOB: Spinal mobilization
RMS: Root mean square
nRMS: Normalized root mean square
SD: Standard deviation
SMT: Spinal manipulative therapy
TES: Thoracic erector spinae
